# Defensive Traits during White Spruce (*Picea glauca*) Leaf Ontogeny

**DOI:** 10.3390/insects12070644

**Published:** 2021-07-15

**Authors:** Antoine-Olivier Lirette, Emma Despland

**Affiliations:** Biology Department, Concordia University, 7141 Sherbrooke West, Montreal, QC H4B 1R6, Canada; a-o.lirette@hotmail.com

**Keywords:** plant-insect interactions, leaf defensive traits, herbivory, spruce budworm, toughness, leaf physical traits, phenology

## Abstract

**Simple Summary:**

Leaves can only toughen after they have finished growing and, as a result, many herbivorous insects specialize in newly developing leaves because softer leaves are easier to chew. The foliage of conifer trees is particularly tough, and so one would expect conifers to invest more defensive chemicals into soft vulnerable growing needles than into tough mature ones. We summarize the literature describing how chemical defenses of foliage change during the growing season in white spruce, an economically important conifer tree. We next report measurements of the toughness of white spruce buds as they swell, burst, and grow into young needles. As expected, buds soften as they swell in spring, but after budburst, needles become tougher until they are similar to previous-year foliage in mid-summer. Leaves grown in the sun are slightly tougher than leaves grown in the shade. However, there was no indication that trees invest more in chemical defense of growing leaves than of mature leaves.

**Abstract:**

Changes during leaf ontogeny affect palatability to herbivores, such that many insects, including the eastern spruce budworm (*Choristoneura fumiferana* (Clem.)), are specialist feeders on growing conifer leaves and buds. Developmental constraints imply lower toughness in developing foliage, and optimal defense theory predicts higher investment in chemical defense in these vulnerable yet valuable developing leaves. We summarize the literature on the time course of defensive compounds in developing white spruce (*Picea glauca* (Moench) Voss) needles and report original research findings on the ontogeny of white spruce needle toughness. Our results show the predicted pattern of buds decreasing in toughness followed by leaves increasing in toughness during expansion, accompanied by opposite trends in water content. Toughness of mature foliage decreased slightly during the growing season, with no significant relationship with water content. Toughness of sun-grown leaves was slightly higher than that of shade-grown leaves. However, the literature review did not support the expected pattern of higher defensive compounds in expanding leaves than in mature leaves, suggesting that white spruce might instead exhibit a fast-growth low-defense strategy.

## 1. Introduction

Physical and chemical changes occur during leaf ontogeny that change palatability to herbivores. Expanding leaves are generally less tough and more nutritious than mature foliage [1,2,3]. This is directly linked to the process of leaf growth: young leaves cannot accumulate lignin, cellulose, or other cell wall components until leaves have ceased growing [4], and as a result, they are both low in toughness and high in nitrogen content. Similarly, sugars and amino acids are translocated to expanding leaves to build tissues. At the end of expansion, leaves change from being a photosynthate sink to a source, and their cells sclerify [5]. The accompanying changes in chemical defense traits remain poorly understood, yet are essential to understanding host tissue choices by insects and the distribution of herbivory damage on plants [1]. Since expanding foliage is both less well mechanically defended and more nutritious, it is historically predicted that plant secondary compounds are maintained at higher concentrations in expanding foliage, to protect this vulnerable stage from herbivores [6]. Furthermore, optimal defense theory predicts high investment in defense of expanding foliage because the same amount of herbivory has more impact on a growing structure than on a fully grown one [1].

Nonetheless, many herbivores still preferentially feed on, and perform better on, expanding foliage. Examples among evergreen plants that retain their leaves over many years include boreal conifers [7,8,9,10], tropical angiosperms [3], and cycads [11]. Ontogenetic changes in foliage quality thus limit the period when the foliage is suitable for herbivorous insects, promoting tight phenological host–herbivore relationships that define a window of opportunity [12,13,14]. The magnitude of these differences between young and old foliage defines the strictness of the window of opportunity for insect herbivores on evergreen trees [15].

White spruce (*Picea glauca* (Moench) Voss) is an economically important resource and a foundation species in North American boreal forests. Its genome has recently been sequenced [16,17], and considerable research is underway to examine the genetic architecture of white spruce traits, including investigating the biosynthesis pathways of defensive compounds [17,18,19] and attempting to select traits for resistance against herbivores [20]. However, the phenology and ontogeny of defensive compound expression in foliage has received little attention, despite the fact that many defoliators on white spruce, including the notorious spruce budworm (*Choristoneura fumiferana* (Clem.)), preferentially feed on expanding foliage [21,22,23]. The window of opportunity for these herbivores has been linked to nutrient content, allelochemicals and toughness [22,24], but contributions of these variables are not clear. In particular, the hypothesis that secondary chemicals should be more concentrated in expanding foliage has not received much attention in spruce.

This paper combines a literature review on the ontogeny of white spruce foliar defense with original research addressing the knowledge gap on seasonal patterns of leaf toughness. We first summarize past work on ontogenetic progression of defense traits in expanding white spruce foliage and highlight knowledge gaps for future research. Review of the literature highlights that toughness is one trait that has received relatively little attention, despite evidence for an important role in defense against the spruce budworm [15]. Therefore, we measured white spruce leaf toughness during the growing season in both expanding and mature foliage in order to improve understanding of the ontogenetic trajectory of mechanical defense during the window of opportunity for herbivores. Leaf physical trait data from this boreal conifer will also contribute to generalize understanding of ontogenetic shifts in plant defense syndromes during leaf development [1].

## 2. Literature Review on Phenology of Spruce Defensive Traits

### 2.1. Leaf Nutritional Traits

Conifer growth in the spring begins before Nitrogen uptake by roots, and therefore depends on remobilization of both Carbon and Nitrogen from reserves stored in one-year-old foliage as they are translocated to developing buds [25]. Indeed, most of the N in new conifer shoots is derived from storage in old needles. Soluble N increases in year-old conifer foliage prior to budburst in spring then quickly drops as N is translocated to developing buds [26,27]. Primary meristems are the source of signal perception for the resumption of growth, responding to both growth regulators and sugars [28].

In spring, before budburst, carbon supply is greater than carbon demand [29], and the carbon needed for primary growth accumulates, mostly in old needles, mainly in the form of starch. Reserves are depleted in the period between budburst and the carbon autonomy of new leaves [28], as sugars are translocated to expanding shoots. In conifers, newly assimilated carbon by older needles is mainly allocated to the canopy during primary growth, with only a minor fraction translocated to the lower stem [30].

Measurement of the elemental composition of white spruce leaves showed that the greatest quantitative changes occurred during the periods of active shoot growth [22]: Total N and P levels were highest in the swelling buds, declined rapidly during initial shoot elongation, and then remained uniformly low and stable through the summer. Levels of K also declined during shoot elongation but did not reach their lowest points until after cessation of shoot elongation. Levels of Ca, Mg, Cu, and Zn in current-year foliage generally decreased during shoot elongation, but then increased afterwards. Total foliar sugar (fructose + glucose + sucrose) in new foliage peaked near the middle of shoot elongation and thereafter generally decreased [22]. To our knowledge, these trends have not been linked to the physiological processes described in the preceding paragraphs, and therefore more research is needed to trace movement of nutrients in expanding shoots with modern methods, and in particular to distinguish between different forms of organic Carbon and Nitrogen only some of which are bioavailable to herbivorous insects [12,31].

### 2.2. Phenolics

Early phenological studies showed that total phenolics in current-year foliage decreased during shoot elongation but increased thereafter: they fell to their lowest levels of 4–5% dw at ca. 80–90% shoot elongation in early June [22]. Mattson et al. [32] similarly reported that total phenolic levels peaked at the time of budbreak in white spruce and declined consistently during shoot growth.

However, total phenolic content is no longer considered a good measure of chemical defense against insects [33]. In more recent years, the structural and functional diversity of spruce phenolics have been much better characterized, and their synthesis from the shikimate pathway is being unravelled [17]. Complex phenolics like lignins clearly play a structural role in spruce leaves [5], but other molecules are thought to defend against fungal pathogens or insect attack. This defensive role is supported by the fact that phenolic synthesis pathways are upregulated in several spruce tissues, including foliage following fungal infection, insect attack, or treatment with the defense hormone methyl jasmonate [17]. Nonetheless, few individual phenolic compounds have convincingly been tested for anti-herbivore activity and the evidence for a defensive role of conifer phenolics remains poorly understood [34]. Some phenolic compounds do accumulate in foliage [17], but to our knowledge, their phenology is unknown except for the two acetophenones discussed below.

Two phenolic compounds have been suggested to play an important role in the defense of coniferous trees against defoliation by the spruce budworm [35,36]. Two sets of acetophenones have been identified from white spruce trees resistant to budworm attack which suffered only light defoliation when other trees around them were heavily damaged: piceol and pungenol (aglycones) and picein and pungenin (their glycosides). The aglycones increase mortality and slow growth in bioassays, but the glycosylated forms appear to have no effect on the budworm [35]. These acetophenones have been shown to be broadly distributed across coniferous trees; in general, the glycosides were found alone or at higher concentrations than the aglycones [36]. Genetic analysis of white spruce trees showed that a glucosyl hydrolase gene, *PgBgluc-1*, was constitutively highly expressed in resistant trees, catalyzing formation of the aglycones from the glycosylated compounds [37]. Levels of both the gene transcripts and the aglycones are highly heritable [36]. In white spruce, the aglycones begin to be expressed in current-year foliage near the end of shoot elongation, when the budworm reach the final instars [36]. These compounds thus do not follow the predicted trend of higher concentration in expanding foliage.

### 2.3. Terpenoids

Terpenoids are considered important defensive compounds of conifers, and their role has been well-studied in defense against stem-boring insects. Oleoresin is comprised of a diverse array of terpenoid compounds mobilized to the site of wounding on stems. Oleoresin can physically ‘pitch out’ or entomb attacking insects as well as clean and seal the wound from microorganisms. In white spruce, induction of a local terpenoid response can be triggered by stem damage and lead to the formation of specialized traumatic resin ducts [18,38].

Terpene Synthase genes exhibit high functional diversity, and differential expression leads to a wide range of different blends [17]. Herbivore damage, pathogen infection or MeJA treatment upregulate terpenoids in different tissues, but it is difficult to disentangle stem vs. leaf responses [39]. Indeed, the ecological role of foliar terpenoids pools is not clear [40]. Several examples show a correlation between conifer foliar terpenoid concentrations and insect resistance [8,41,42]: in particular, spruce terpenoids have been suggested to be implicated in natural resistance against both the eastern and western spruce budworm [43]. However, manipulative experiments with bioassays included varying monoterpene concentrations give ambiguous results on the performance of folivores [32,44].

Emission of terpenoid volatiles can be induced in conifer foliage following insect damage [40,45,46]; these volatile compounds may attract natural enemies, but their direct effect on folivores is not clear. However, increases in synthesis of volatile monoterpenes do not necessarily lead to increases in foliar pools because of increased emissions [40,46]. Foliar monoterpene pools are at a metabolic crossroads, between synthesis, emission, storage and translocation to other tissues and may not be under selective pressure from folivores [18]. More research is therefore needed to characterize the patterns and phenology of terpenoid synthesis, emission, and translocation both into and out of leaves.

A few studies have characterized the time course of monoterpene foliar pools: white spruce foliar monoterpenes increased over the growing season in a warm, dry year but stayed low in a cooler, wetter year [47]. A similar seasonal increase has previously been observed in white spruce [48] and other conifers [45]. In Norway spruce (*Picea abies* (L.) H. Karst, 1881), expression of a terpene synthase gene was shown to be low in buds and to increase during bud swelling and shoot elongation [17].

Examination of individual monoterpenes showed a spring peak in d-3-carene, followed by a gradual increase in bornyl acetate over the summer [47]. D-3-carene is of particular interest as it is similarly expressed only during leaf expansion in Scots pine (*Pinus sylvestris* L. [49]) and defines two chemotypes of this species. Moreover, high and low levels of d-3-carene in growing shoots define Sitka spruce (*Picea sitchensis* (Bong.) Carrière) genotypes resistant and susceptible to white pine weevil [50,51]. This compound seems the best candidate among monoterpenes for a defensive effect in conifer foliage.

### 2.4. Alkaloids

Several 2,6 disubstituted piperidine alkaloids have been detected in the wood, bark, roots and foliage of conifers; in *Picea*, the two main ones are *cis*-pinidinol and epidihydropinidine [52,53]. The function of these is still uncertain; they might be involved in winter hardiness or defense against pathogens, but they are thought to deter herbivores. Some evidence suggests that *cis*-pinidinol and epidihydropinidine deter feeding by spruce budworm larvae and oviposition by female moths [54,55].

In Norway spruce, total alkaloid concentrations peak at budburst and decrease during shoot elongation. However, the peak is composed mostly of accumulation of precursor compounds used in biosynthesis of *cis*-pinidinol and epidihydropinidine, and the concentrations of these two main compounds remain stable in mature foliage, including multi-year old foliage [56].

Concentrations of these alkaloids are low compared to those of phenolics and terpenoids and their role in spruce metabolism or defense has received little attention [57,58].

### 2.5. Toughness

Toughness is a highly effective defense against insect herbivores that reduces the nutritional value of the leaf and presents mechanical problems for chewing insects [59]. In evergreen tropical forest plants, toughness is a strong predictor of herbivory rates, and most leaf damage occurs during leaf expansion before maximal toughness is achieved [60,61]. Foliar toughness is particularly problematic for young larvae that do not have the mandibular morphology necessary to pierce tough tissues [62,63,64].

Mature needle toughness has been suggested to determines the window of opportunity for insect herbivores on boreal conifers [15], yet has seldom been measured empirically. Foliar toughness was the most likely explanation for the inability of young larvae of hemlock looper (*Lambdina fiscellaria fiscellaria* (Guenée)) to initiate feeding on old foliage of eastern hemlock (*Tsuga canadensis* (L.) Carrière) [7]. Similarly, young larvae of the palewinged gray moth (*Iridopsis ephyraria* (Walker)) exhibited high mortality when feeding on old foliage of balsam fir (*Abies balsamea* (L.) Miller) and their inability to feed was attributed to leaf toughness [65]. Toughness of mature foliage is an important mechanism inhibiting initiation of feeding by emerging eastern spruce budworm on black spruce (*Picea mariana* (Mill.) B.S.P.) [15,24]. Although it is well understood in tropical angiosperms, the role of leaf toughness in concentrating herbivore damage on young expanding conifer foliage deserves further attention. Therefore, we next present results from original research on the seasonal pattern of toughness of both mature and expanding white spruce foliage in order to test the hypothesis that expanding foliage is less tough than mature leaves.

## 3. Materials and Methods

Leaf water content and toughness were measured at weekly intervals from 24 May to 26 July 2018 (N = 10 measurement dates) on 12 white spruce trees at the Valcartier Forestry Research Station Arboretum Serge Légaré (latitude, 46.95° N; longitude, 71.48° W; elevation, 152 m). These trees represented 3 clones each of 4 genotypes. Branches were selected based on the presence of buds and year-old needles and were always taken from the bottom half of the crown ca 1.5–2 m above the ground. The trees used in this experiment were from seed lot C9612893, which was the result of an intra-provenance cross performed in the white spruce hybridization parc “Parc Algonquin” at Cap Tourmente, Québec. The female clone (ALG-10) in the cross was found at a latitude of 45.50° N and a longitude of 78.30° W at an elevation of 400 m and the male clone (ALG-8) at a latitude of 45.50° N and longitude of 78.30° W and an elevation of 400 m. The selected trees (6M, 1A, 6O, 12F, 6S, 2F, 12A, 1C) were the result of somatic embryogenesis as in [66], from the seed lot C9612893.

Toughness was measured with a penetrometer [15,24] on 10 individual needles and 10 buds per tree and these were pooled to create one value per tree per date for both needle and bud toughness. The penetrometer consists of a modified microscope with removed lenses to which a Medio-Line Spring Scale (Pesola AG, Baar, Switzerland) is affixed, reversed in a way that pushing instead of pulling will yield a measurement. A syringe head is attached to the end of the measuring device facing towards the needle or bud. Toughness is then measured by slowly raising the scale until the syringe head pierces the needle or bud and the measurement is read from the scale and recorded. Single needles or buds were plucked from freshly cut branches with the cut end kept in water before being placed on the penetrometer. To ensure consistency between the readings, the syringe needle used to pierce through the spruce needles and measure their toughness was changed every 5 readings; any more was determined to dull the edges of the needle and affect the toughness reading. Groups of 10 needles were weighed on a Sartorius analytical balance (0.01 mg accuracy), then air-dried at 60 °C for 48 h, and water content was measured as the difference between wet and dry weight. Needles were weighed in groups due to their low mass.

Measurements were taken on previous-year foliage and expanding buds or foliage. At each sampling date, buds were sampled for toughness measurement and bud stage was recorded according to the scale developed by [67] and illustrated in this field guide [68]. The scale is numerical from 0 to 6 each representing a stage of development of the bud, which can be assessed visually as follows: (0) closed and dormant buds, (1) Bud scales are opening, and a white spot is visible at the top of the bud, (2) buds are elongating, (3) buds are swelling, (4) buds scales are translucent, and the needles are partly visible, (5) bud scales are ripped at the base of the bud and needles are tightly bundled, (6) needles are elongating and expanding laterally. After buds reached stage 6 (buds fully open), expanding needles were sampled instead. Current-year measurements were considered as buds (stages 0–6) from 24 May to 4 June, and as expanding needles from 28 June to 26 July. Data from 7 June to 21 June (N = 3 measurements) were omitted from analyses including current-year foliage because the opening buds were too soft for meaningful measurement.

A mixed model was run in R including individual tree as a random factor, and foliage type (previous year foliage, expanding bud or current-year foliage), genotype, measurement date (as a Julian date) and water content as fixed factors. Model simplification was based on AIC. Relationship between toughness and water content and between toughness and Julian date was estimated with Pearson correlations. Genotype was included as a variable in our model as a potential explanation for the difference in leaf toughness but did not contribute significantly to the model.

To test for potential environmental effects on leaf toughness, toughness was also compared between 10 previous-year sun-exposed and shade leaves on the same 12 trees on one date in mid-July. Shade leaves were defined as leaves on branches in the lower crown that were continuously in the shade, whereas sun leaves were leaves in the lower crown that received at least 4 h direct sunlight daily. Leaf sampling was done during those hours of sun exposure on a sunny day. The effect of sun exposure on leaf toughness was estimated with a mixed linear model including individual tree as a random factor and genotype and sun exposure as fixed factors.

## 4. Results

The model including foliage type, Julian date, the interaction between the two, and water content (AIC = 1426) was retained by model simplification over the full model including also genotype (AIC = 1489). The significant interactions between foliage type and Julian date (current year foliage * Julian date coefficient: 5.02, *p* < 0.0001; previous year foliage * Julian date: 3.42, *p* < 0.001) imply that the trajectory of toughness over the growing season differs between swelling buds, current year foliage and previous year foliage and thus that main effects cannot be directly interpreted. The effect of water content on leaf toughness was not quite significant (0.5573, *p* = 0.06), but its removal did not improve the model.

**Figure 1:**
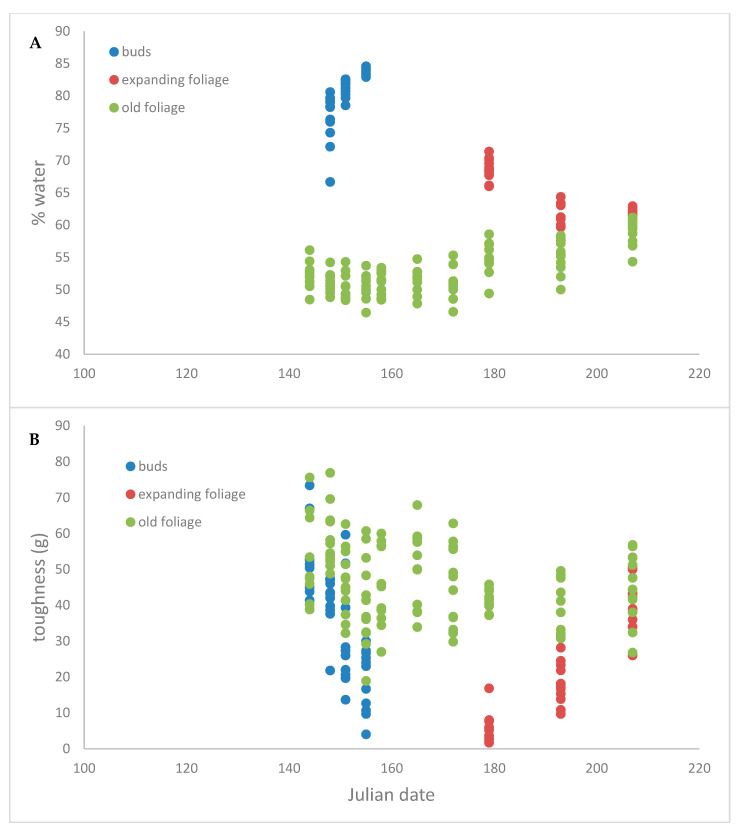
Time course of white spruce (**A**) water content and (**B**) leaf toughness. Green represents previous-year needles, blue represents swelling buds and red the foliage that emerges from those buds. There is a two-week gap between measurement of buds and expanding foliage in which the opening bud was too soft to permit toughness measurement.

Figure 1 shows how bud toughness decreases as the buds swell (stage 2 to 6), the gap between the bud measurements and the expanding foliage measurements is due to the first few weeks after the buds open and the foliage was too soft for meaningful measurement. After budburst, the toughness of expanding foliage gradually increases until it is similar to that of previous-year foliage at the end of expansion.

**Figure 2:**
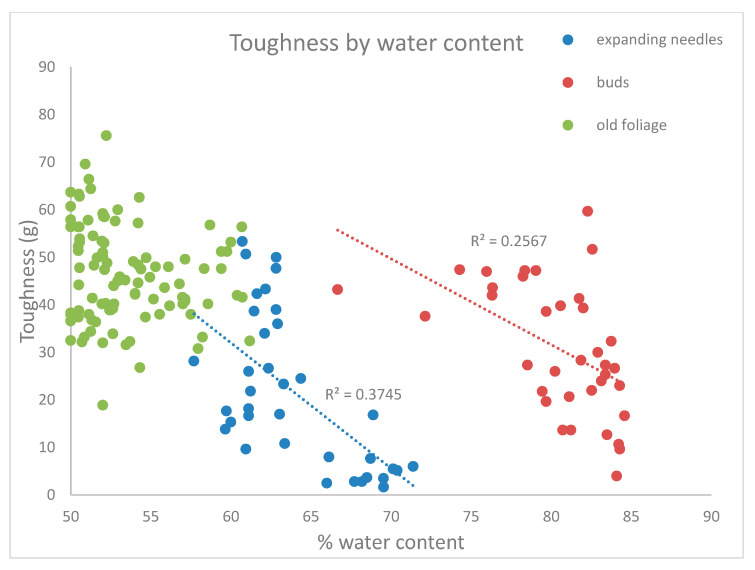
Regressions of toughness against water content. Regression lines and R^2^ values are shown for expanding foliage and for buds, but not for mature foliage because the relationship was not statistically significant.

Pearson correlations showed that toughness of mature foliage decreased slightly during the season (Pearson correlation coefficient: −0.251; *p* = 0.006) but was not related to water content (Pearson correlation coefficient: −0.081; *p* = 0.37)—see Figure 2. The toughness of expanding buds also showed a negative relationship with Julian date (Pearson correlation coefficient: −0.64; *p* < 0.0001), and decreased with water content (Pearson correlation coefficient: −0.506; *p* = 0.0016) as well. Current-year leaves toughness increased as the season progressed (Pearson correlation coefficient: 0.96; *p* < 0.0001), but decreased with water content (Pearson correlation coefficient: −0.61; *p* < 0.0001).

**Figure 3:**
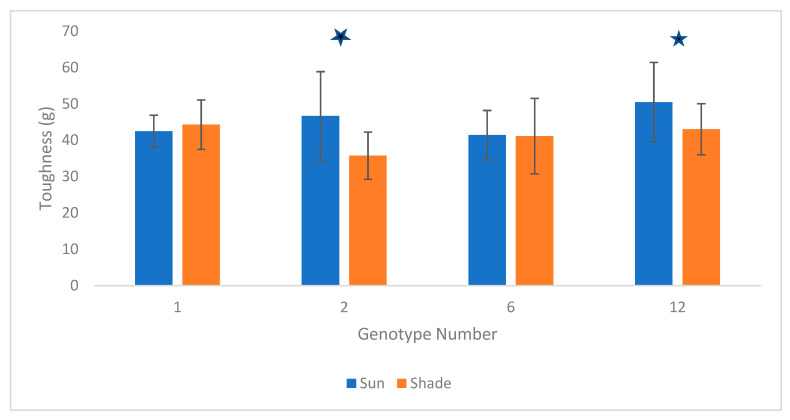
Average (+/− standard deviation) toughness of sun vs. shade leaves in four different white spruce genotypes. The numbers 1, 2, 6, and 12 represent the different genotypes of clones chosen at the Arboretum for this study. Stars indicate the two genotypes for which significant differences between sun and shade foliage were observed.

The mixed model comparing toughness between sun leaves and shade leaves showed a significant interaction between sun exposure and genotype, showing that sun leaves were tougher than shade leaves in 2 of the 4 genotypes (coefficient: 12.733; *p* < 0.0001; coefficient: 9.27; *p* < 0.0001)—see Figure 3. Nonetheless, an overall paired t-test showed that sun leaves are significantly tougher than shade leaves (t = −3.55, df = 231.08, *p*-value = 0.0004). Pearson correlation showed no correlation between leaf toughness and water content (Pearson correlation coefficient: 0.089; *p* = 0.68).

## 5. Discussion

Our results show that leaf expansion in white spruce thus generates the predicted window of opportunity in terms of providing less tough foliage for a short period before growth is completed. Review of the literature suggests that nutritional content, mostly Nitrogen content, also decreases with leaf expansion, but that patterns of defensive chemistry are less clear. Indeed, some compounds decrease as expected with leaf expansion (e.g., total phenolics, alkaloids, d-3-carene), while others, by contrast, increase (e.g., total monoterpenes, acetophenones). However, the defensive role of these compounds remains poorly defined, especially given that many specialist herbivores, including the spruce budworm, possess effective detoxification enzymes [69]. It must be noted that many of these compounds could also be implicated in plant metabolism in roles other than anti-herbivore defense and these other roles might determine their seasonal time course.

As expected, toughness of expanding foliage was inversely proportional to water content. However, water content was a poor predictor of toughness of mature foliage, and this is consistent with a previous study that links conifer toughness to fiber and particularly to hemicellulose content in balsam fir and black spruce [15]. Previous work [22] showed that water content of current-year white spruce foliage peaks soon after budbreak in mid-May as water flows in for cell expansion [26], and then steadily decreases during the shoot growth period until mid-July. Toughness of the new needles increased rapidly during the period of shoot elongation as water content dropped [22].

Sun leaves were found to be slightly tougher than shade leaves, similar to tropical angiosperms where the high leaf specific area of sun leaves contributes to higher toughness [70]. However, this effect only attained statistical significance in two of four genotypes, and effect size was smaller than that of leaf phenology. It nonetheless suggests that sun exposure should be controlled for when estimating foliage toughness in conifers.

During leaf ontogeny, cells continue to grow in size after cell division, mostly via expansion of a water-filled intracellular vacuole. This growth is accompanied by the development of a primary cell wall, but the sclerification and thickening of the secondary cell wall that increases toughness and strengthens the leaf only begins after the cell has reached its final size [4]. Plant cell walls are made of long cellulose microfibrils whose spatial organization in a matrix of polysaccharides (e.g., hemicellulose), proteins, and phenolics (e.g., lignin) promotes toughness [71]. The thickness and chemical composition of cell walls is variable, and thick cell walls rich in hemicellulose and lignin generally imply higher biomechanical strength and toughness [71]. Spruce needles comprise three tissue layers: the protective dermal tissue includes the epidermis and hypodermis and is overlain by a heavy waxy cuticle, the mesophyll is the bulk of the needle where photosynthesis occurs and contains two resin canals, and the vascular tissue in the center is surrounded by an endodermis [72]. In a mature spruce needle, sclerified thick-walled cells are found in the single-layer epidermis, in the hypodermis, near the resin canals and in the endodermis [5]. The mechanism of toughening during white spruce leaf ontogeny is not clear, but likely includes thickening and strengthening of secondary cell walls in the hypodermis and possible in the endodermis [4].

Despite the increase in toughness in developing white spruce leaves that we demonstrate here, the literature does not provide clear evidence for the compensatory higher investment in chemical defense of young leaves. Total phenolics appear to follow the expected trends, but the focus on total phenolics is out-of-date, as these compounds are very diverse and their effects on the alkaline Lepidopteran midgut depends on their chemical structure [33]. The best characterized phenolic compounds, the acetophenones picein and pungenin show the opposite pattern and only accumulate in foliage once it matures [19]. The time course of terpenoids has not received much attention with modern methods; what little evidence exists suggests that overall monoterpene concentration increases in maturing foliage (against the expected trend), but that one compound, d-3-carene, shows the predicted trend of greater concentration in expanding foliage and hence might warrant further attention [47].

An alternative strategy for leaf defense during expansion involves investing in fast growth to escape the vulnerable stage, and investing in defense later [73]. Indeed, growth and defense functions are often considered to trade-off in plants [6,74], and one expression of this trade-off occurs in leaf ontogeny where plants can invest either in rapid leaf growth to attain the defended mature stage quickly or in defense of slower growing expanding leaves. In general, caterpillars grow faster on species with fast-expanding than slow-expanding leaves, but have less time to complete their development before the leaf matures and defenses set in [73]. White spruce has been shown to exhibit faster leaf expansion than related species [32,75], suggesting that it exhibits the fast growth strategy and might invest less in defenses of expanding foliage. White spruce is considered a good host for several defoliators, the best-studied of which is the spruce budworm, but escapes too heavy foliage loss because of fast foliage growth [76]; these are both traits consistent with a fast-growth low-defense strategy.

A recent meta-analysis of globally important insect pests suggests that many show responses to climate change that will lead to increased damage [77]. Phenological synchrony is increasingly recognized as important in plant-insect interactions, including in the population dynamics of outbreaking forest pests, as both host plant characteristics and insect requirements change over their respective ontogenies [12,31,78,79]. Understanding ecology and trophic relationships under a changing climate requires a focus on the ontogenetic and physiological underpinnings of phenology of the interacting partners [80]. Plant phenology is usually considered in terms of visible changes, like budburst, but phenological traits that are fundamental to plant relationships with herbivores are often ‘cryptic’, or not visible to the human observer, and hence much less well understood. These cryptic traits are increasingly under molecular and genomic investigation [17,18], and studies need to consider the phenology of these traits. Investigation of cryptic phenologies, like the toughness, chemical and nutritional content traits covered here, is essential to understand plant-mediated effects on herbivores, including the spruce budworm [81].

## Data Availability

Data are available at Concordia University’s online data repository, Spectrum.
